# Anger or pride? The effect of overqualification on employees’ first job behavior from the perspective of the proactive motivation model

**DOI:** 10.3389/fpsyg.2025.1682877

**Published:** 2026-01-05

**Authors:** Geng Li, Ziyi Zhang, Chunxin Zhang, Junqi Dou

**Affiliations:** College of Management and Economics, Tianjin University, Tianjin, China

**Keywords:** perceived overqualification, proactive behavior, work anger, authentic pride, perceived supervisor support

## Abstract

**Purpose:**

This study investigates the relationship between perceived overqualification and proactive behaviors in the first job, focusing on the mediating roles of job anger and authentic pride and the moderating effect of perceived supervisor support. The research aims to address the challenge of maintaining and stimulating proactive behaviors among overqualified first-time employees.

**Design/methodology/approach:**

Based on the proactive motivation model, this study collected data from 695 first-time employees and analyzed the relationships between perceived overqualification, emotions, proactive behaviors, and perceived supervisor support using statistical methods.

**Findings:**

The results indicate that perceived overqualification negatively affects proactive behavior. Job anger and authentic pride serve as significant mediators in this relationship, while perceived supervisor support moderates the effect of perceived overqualification on proactive behavior.

**Originality:**

This study contributes to the literature by integrating emotional mediators and supervisory factors into the analysis of proactive behavior among overqualified employees. It provides practical insights for enhancing human resource management and fostering proactive behaviors in the workplace, particularly for first-time employees facing overqualification.

## 1 Introduction

The stagnation of global economic development and the widespread access to higher education have intensified competition in the labor market. An increasing number of highly qualified individuals are engaging in jobs that fall below their knowledge, skills, and experience levels-a phenomenon known as “overqualification.” Individuals’ subjective perception of their own overqualification is referred to as perceived overqualification (POQ) (Maynard et al., 2006).

Recent graduates generally possess strong comprehensive qualities and adaptability, coupled with lower labor costs (Wang, 2020), making them particularly attractive to employers during campus recruitment for first-time job seekers. However, the relatively high qualifications of this group can lead to a greater likelihood of perceived overqualification in their first job. First, individuals entering their first employment are at the initial stage of their careers, during which their career identity, career expectations, and future work self are rapidly forming. They tend to have clear and strong expectations that their positions will allow them to utilize their learned skills, provide developmental opportunities, and offer visible career trajectories. Therefore, when person–job misfit occurs, the gap between expectations and reality is more likely to be activated, eliciting pronounced psychological reactions (Takeuchi et al., 2021; Guan et al., 2014). Second, newcomers typically possess weaker organizational resources and bargaining power—they rely more heavily on the organization’s training, guidance, and embedded resources to achieve successful career socialization. Consequently, any early experience of being undervalued or having their skills underutilized can more sensitively affect their emotions, career self-efficacy, and organizational commitment (Cai et al., 2023; Batistič and Kaše, 2022). Third, during the initial employment stage, socialization relationships are not yet well established. External support (such as supervisor support) and organizational socialization practices exert stronger shaping effects on newcomers’ career adaptation and proactivity (Yang et al., 2024; Takeuchi et al., 2021). In summary, within the first-employment context, perceived overqualification not only tends to negatively affect individuals’ emotions and behaviors (Yang and Li, 2021), but may also influence their career adaptation resources and relational networks, leading to more enduring or amplified behavioral consequences compared with senior employees.

The first job represents a critical juncture in graduates’ careers, significantly influencing their future career trajectories. For organizations, the perceived overqualification of individuals in their first job will reduce the stability of employees, increase the cost of the enterprise, and highlight deficiencies in job design, qualification and recruitment standards (Martinez et al., 2014). Therefore, it is important to study the phenomenon of overqualification of first-time employees to improve the level of human resource management in organizations and promote the growth and development of employees.

To adapt to the ever-changing external environment, more organizations expect employees to have spontaneous, change-oriented and future-oriented behaviors, i.e., proactive behaviors (Li, 2020), which are conducive to both organizational development and individual growth. Therefore, when individuals feel overqualified in their first job, stimulating their proactive behavior becomes crucial.

Regarding the relationship between perceived overqualification and proactive behavior, academic findings remain inconsistent. Some studies have found a positive relationship between perceived overqualification and proactive behavior (Zhang et al., 2016), whereas a larger body of research suggests that the higher the perceived overqualification, the less proactive behavior is exhibited (Li et al., 2021; Wu et al., 2017). However, there remains a gap in research on this relationship in the context of first-time jobs. Given that there may be underlying mechanisms between perceived overqualification and proactive behavior that have not been fully explored, this study aims to construct a comprehensive model to deeply explore and clarify the complex relationship between these two factors in the context of a first job.

The proactive motivation model proposed by Parker is an important theory for understanding the emergence of proactive behavior. According to the model, situational variables, as distal factors, can influence proactive behavior through three different motivational pathways, namely “ability,” “willingness,” and “emotion” (Parker et al., 2010). The emotional pathway, which operates through emotional arousal and action energy, constitutes a key mechanism for explaining how external contextual factors shape proactive behavior. Parker suggest that not only positive emotions can stimulate intrinsic motivation and promote proactive behavior, but also negative emotions (e.g., negative emotions reflecting the gap between ideals and reality) can be the motivation for triggering proactive behavior (Parker et al., 2010).

However, prior research applying the proactive motivation model to explore the emotional pathway has often treated emotion as a unidimensional construct of positive versus negative affect, overlooking the distinctions among discrete emotions in terms of action tendencies, resource mobilization, and cognitive appraisal (Peng and Wu, 2021). Recent studies suggest that such oversimplification obscures critical mechanisms. In organizational contexts, different negative and positive emotions do not influence proactivity equivalently. For instance, anger can, on the one hand, drive corrective or confrontational actions when individuals perceive injustice (Porat and Levy Paluck, 2024); on the other hand, it may deplete cognitive resources and trigger defensive motivation, thereby leading to behavioral withdrawal (Zhang et al., 2024). In contrast, authentic pride, as a positive emotion rooted in internal attribution, typically enhances self-efficacy, broadens cognitive scope, and strengthens goal commitment, thus fostering exploratory and sustained proactive behavior (Schilpzand et al., 2025). Meanwhile, emerging research on perceived overqualification has further revealed its dual nature. When resources, support, or self-activation mechanisms are present, POQ may promote proactive and innovative behaviors by enhancing role-breadth self-efficacy or stimulating creative self-expression (Lu et al., 2023). Conversely, under conditions of resource scarcity or heightened deprivation, POQ is more likely to evoke anger, exhaustion, and avoidant behavior through perceptions of relative deprivation or unfairness (Liu et al., 2024).

Therefore, the present study selects work anger and authentic pride as two specific emotional mediators to explain the mechanism through which perceived overqualification affects proactive behavior. The former represents energy depletion and defensive tendencies under conditions of injustice or deprivation appraisal, whereas the latter reflects self-efficacy activation and promotive motivation in supportive or achievement-feedback contexts.

In addition, prior research has identified perceived supervisor support (PSS) as an important antecedent of perceived organizational support (POS) (Rhoades and Eisenberger, 2002) and the most immediate source of social resources that employees experience in the workplace (Pan and Hou, 2024). Moreover, perceived organizational support may moderate the relationship between perceived overqualification and individual behaviors (Liu and Wang, 2012). On the other hand, in studies related to proactive behavior, the supportive behavior of superiors has received attention by being proven to be an important factor influencing employees’ proactive behavior (Erdogan et al., 2011; Cai et al., 2019; Pan and Hou, 2024). For employees entering the workforce for the first time, the lack of stable organizational status and accumulated experience makes them significantly more dependent on their supervisors’ feedback, recognition, and guidance (Yang et al., 2024). However, existing studies have mostly explored the moderating variables affecting the relationship between perceived overqualification and employee behavior from the perspective of sense of organizational support, while neglecting the role of perceived supervisor support. Therefore, this study introduces perceived supervisor support as a moderating variable to explore the boundary conditions of the influence of perceived overqualification on proactive behavior.

In summary, this study examines the context of initial employment and develops a mechanism based on the proactive motivation model, focusing on the “overqualification—emotion—behavior” pathway. It explores how perceived overqualification influences proactive behavior through different types of emotions. Additionally, the study introduces the moderating effect of supervisor support within the emotional pathway, highlighting the boundary role of supervisor support in emotional regulation and behavior maintenance.

## 2 Theory and hypotheses

### 2.1 Proactive behavior

According to social cognitive theory, human beings possess self-regulatory and goal-directed capabilities that enable them to proactively evaluate external environments and act accordingly. Individuals with long-term goals tend to shape situations in advance to achieve desired outcomes (Frese and Fay, 2001; Ayed et al., 2024; Zhou, 2024). Proactive behavior is now defined as self-initiated and future-oriented actions that employees take to bring about change and improvement in themselves or in their organizational environment (Fu et al., 2024).

Previous research has primarily focused on the factors influencing proactive behavior (Cai et al., 2019), with individuals’ knowledge, skills, and experience being key predictors. Fay and Frese found that employees with higher qualifications are more likely to engage in proactive behaviors (Frese and Fay, 2001). Emotions also play a significant role: positive emotions enhance proactive behavior by improving employees’ attitudes and optimism (Mehmood et al., 2024; Fritz and Sonnentag, 2009), while negative emotions, such as those arising from psychological contract breaches, tend to diminish proactive behavior (Bal et al., 2011). However, Bindl and Parker argued that both positive and negative emotions can activate proactive behavior, depending on whether the emotions are in an “activated” state (Parker et al., 2010).

One important outcome of this research is the proactive motivation model proposed by Parker and other scholars. The model suggests that proactive behavior is influenced by three pathways: the ability pathway (individuals’ assessment of their ability to initiate proactive behavior), the willingness pathway (individuals’ perception of the value of the behavior), and the emotional pathway (The motivational role of emotion in action intentions and behavioral execution) (Parker et al., 2010). In recent years, scholars have begun to focus on the relatively overlooked emotional pathway within the proactive motivation model, emphasizing the crucial role of discrete emotions (such as anger, fear, pride, etc.) in the process of generating proactive behavior; meanwhile, although recent studies on POQ have gradually explored mediating mechanisms such as emotions and exhaustion, empirical research that systematically incorporates discrete negative and positive emotions into the emotional pathway of the proactive motivation model remains limited. Specifically, few studies have simultaneously examined their parallel mediating effects on employees’ proactive behavior (Lu et al., 2023; Liu et al., 2024; Abdalla et al., 2024; Porat and Levy Paluck, 2024).

Drawing on the proactive motivation model, this study focuses on the emotional pathway to examine how perceived overqualification influences proactive behavior among first-time employees. Work anger and authentic pride are selected as parallel mediators to capture the bidirectional emotional mechanisms underlying proactive action. This research not only extends the theoretical scope of the proactive motivation model by incorporating discrete emotions, but also sheds light on the psychological responses and behavioral regulation logic of newcomers under perceived misfit situations.

### 2.2 Perceived overqualification and proactive behavior

Overqualification occurs when individuals possess more skills, knowledge, education, or experience than necessary for their job (Erdogan et al., 2020; Erdogan and Bauer, 2021). It can be viewed objectively, as a mismatch between qualifications and job requirements, or subjectively, as the employee’s perception of being overqualified (Erdogan et al., 2011; Khan et al., 2022). The relationship between objective overqualification and perceived overqualification was significant (Pan and Hou, 2024). In addition, perceived overqualification is generally seen as a better predictor of employee psychology and behavior than objective overqualification (Li et al., 2021; Hu et al., 2015; Lu et al., 2023). For employees entering the workforce for the first time, this psychological experience tends to be particularly salient. New entrants often possess relatively high levels of education and achievement expectations, yet their work roles remain unstable and their professional identities are still under construction (Li and Horta, 2024). When they perceive that their abilities are not being fully utilized, they are more likely to experience psychological imbalance and motivational conflict, which in turn undermines their proactive behavior.

In terms of work-related behaviors, plentiful studies have found that perceived overqualification can have a number of negative effects on employees (Li et al., 2021). For example, perceived overqualification has been found to significantly increases employees’ turnover intentions (Harari et al., 2017), trigger counterproductive work behaviors (Fine and Edward, 2017), and reduce pro-organizational voice behaviors (Frese and Fay, 2001), in which anger (Liu et al., 2015) and work boredom (Kim et al., 2021) play a mediating role.

Conversely, there is a scarcity of studies examining the impact of perceived overqualification on positive behaviors, and the findings are somewhat contentious. Some studies have found that perceived overqualification negatively affects individuals’ positive behaviors; Erdogan found that perceived overqualification was negatively related to helpful and constructive behaviors at work (Erdogan et al., 2020), and Kim found that perceived overqualification promotes boredom at work, leading to a decrease in organizational citizenship behaviors (Kim et al., 2021). Another part of the research found that perceived overqualification facilitates positive behaviors among employees. Zhang found that employees with higher perceived overqualification produce higher role breadth self-efficacy at work, which in turn produces more proactive behaviors (Zhang et al., 2016). It has also been shown that when employees with higher perceived overqualification perceive stronger organizational support, they engage in more innovative behaviors (Luksyte and Spitzmueller, 2016). Although studies on the relationship between perceived overqualification and proactive behavior have focused on specific behaviors such as advice, organizational citizenship behavior, and innovative behavior, few scholars have studied proactive behavior as a whole concept, which may lead to less than comprehensive and accurate research results. Therefore, it is necessary to empirically explore the relationship between perceived overqualification and the overall concept of proactive behavior.

Moreover, these divergent findings suggest that the influence of perceived overqualification on proactive behavior is essentially an emotion-driven process rather than a unidirectional cognitive reaction. For first-time employees whose self-efficacy and career beliefs are not yet firmly established, emotional responses play a particularly critical role. They tend to evaluate the worthiness of work engagement through their emotional experiences: when they perceive job–person misfit as a sign of their abilities being overlooked or their growth being constrained, negative emotions and psychological withdrawal are more likely to arise.

From the perspective of social cognitive theory, individuals continuously regulate their behavior based on self-efficacy and environmental feedback. When newcomers perceive themselves as “overqualified” they tend to reassess the congruence between self-value and external rewards, a cognitive discrepancy that may elicit negative emotions such as frustration and anger. Thus, perceived overqualification is not merely a matter of skill–job mismatch, but a psychological state that influences proactive behavior through emotional pathways.

Integrating the proactive motivation model with social cognitive theory, this study proposes that perceived overqualification affects first-time employees’ proactive behavior through its impact on emotional experiences—specifically, work anger and authentic pride. A higher level of perceived overqualification intensifies feelings of resource underutilization and perceived unfairness, thereby diminishing proactive behavior.

*Hypothesis 1*: Perceived overqualification negatively affects proactive behavior.

### 2.3 Mediating role of work anger

Work anger is a strong negative emotion triggered by work factors in a work situation, which is mainly manifested as dissatisfaction, irritation and exasperation toward work aspects (Zhang et al., 2019; Namaziandost et al., 2023).

According to social cognitive theory, individuals interpret and evaluate their environment based on self-efficacy and external feedback. When first-time employees—who are in the early stage of constructing their professional identity—perceive a mismatch between their qualifications and job requirements, particularly when they believe their knowledge and abilities are underutilized, they are likely to experience a “low-control–high-investment” cognitive imbalance. This imbalance tends to be interpreted as “wasted effort” or “unrecognized competence” which, in turn, elicits feelings of anger.

Within the proactive motivation model, the emotional pathway determines whether individuals are willing to transform their cognition and abilities into actual extra-role actions. Negative and high-arousal emotions often narrow cognitive scope and deplete self-regulatory resources, thereby weakening the cognitive and emotional energy required for problem identification, planning, and implementation (Parker et al., 2010). When first-time employees experience perceived overqualification and interpret it as an indication of “wasted ability” or “unfair return” such evaluations are likely to be emotionally translated into feelings of anger or deprivation (La and Yun, 2019), which subsequently trigger work anger.

In summary, perceived overqualification triggers employees’ work anger, and considering that negative emotions tend to hinder positive behaviors, individuals who feel overqualified may reduce proactive behaviors due to increased work anger. Therefore, this study proposes:

*Hypothesis 2*: Work anger mediates the process of perceived overqualification affecting proactive behavior.

### 2.4 Mediating role of authentic pride

Pride is an emotional state in which the sense of self is enhanced by others’ recognition of one’s achievements, including two subtypes: authentic pride and arrogant pride. Authentic pride stems from internal, controllable and unstable attributions (e.g., effort) usually leading to positive outcomes, whereas arrogant pride stems from internal, stable and uncontrollable (e.g., talent) attributions usually leading to negative outcomes (Tracy and Robins, 2007). This paper focuses on authentic pride.

According to social cognitive theory, individuals’ self-efficacy and emotional experiences depend on their cognitive processing of environmental feedback (Bandura, 1977). When a work context allows individuals to attribute their achievements to personal effort, it tends to elicit positive emotions such as authentic pride. However, the emergence of such positive emotions relies on two critical conditions: first, whether the task is sufficiently challenging to warrant personal effort; and second, whether the organizational environment provides corresponding feedback and recognition (Bandura and Cervone, 1986).

For first-time employees, these two conditions are often difficult to meet. When they perceive that their abilities far exceed job requirements, they may possess objectively strong competence but still find it hard to attribute their performance to “growth through effort” due to repetitive tasks, limited feedback, and low autonomy. Consequently, they are more likely to feel that their abilities are being underutilized or undervalued (Zhang et al., 2022). Such “unchallenging success” does not evoke authentic pride; instead, it may lead to a hollow sense of accomplishment or psychological fatigue (Holbrook et al., 2014). Conversely, when first-time employees receive recognition from their supervisors and are provided with opportunities to apply their skills, perceived overqualification may transform into a positive efficacy belief that enhances authentic pride (Luksyte and Spitzmueller, 2016).

Authentic pride emotions may influence proactive behavior. Positive emotions broaden the scope of an individual’s attention and increase the flexibility and inclusiveness of an individual’s thinking, leading individuals to believe that they can engage in a wider range of jobs, which in turn enhances the individual’s tendency to engage in proactive behavior (Simon et al., 2019). Moreover, proactive behavior is also a risky behavior, and positive emotions lead to a more positive mindset and more optimistic expectations about the outcome of proactive behavior (Fritz and Sonnentag, 2009), which makes it more likely that proactive behavior will be taken, and thus individuals in an authentic pride mood may be more willing to attempt this behavior (Williams and DeSteno, 2008).

In summary, perceived overqualification leads to a depletion of employees’ authentic pride, and considering that positive emotions often accompany positive behavioral outcomes, individuals who feel overqualified may be less likely to perform proactive behaviors due to the undermining of authentic pride.

*Hypothesis 3*: Authentic pride plays a mediating role in the process of perceived overqualification affecting proactive behavior.

### 2.5 Moderating effect of perceived supervisor support

Perceived supervisor support is an overall perception of employees that their supervisors value their work achievements and pay attention to their salary and benefits package, promotion and development (Eisenberger et al., 2002).

According to social cognitive theory, individuals’ self-efficacy and emotional experiences largely stem from social feedback and vicarious reinforcement. For first-time employees who lack a stable professional identity and well-established self-efficacy, supervisors serve as the most direct sources of social feedback and resource support within organizations. Their attitudes and behaviors critically shape how newcomers interpret external events and form self-evaluations (Bauer et al., 2007).

In the context of initial employment, perceived overqualification is easily interpreted as “underestimated ability” or “unrecognized effort” which may evoke frustration and work anger. When supervisors provide clear task guidance and recognition-based feedback, they help employees reframe the meaning of their work, alleviating perceptions of unfairness and mitigating resource depletion, thereby reducing the emergence of anger (Xiao et al., 2022). Conversely, a lack of support tends to amplify this sense of imbalance, making anger more likely to translate into withdrawal or passive behavior. At the same time, when individuals experience high levels of perceived supervisor support, their authentic pride derived from personal competence and effort is positively reinforced. Supervisory recognition, resource provision, and developmental opportunities encourage employees to channel their pride into proactive behavior. In contrast, when supervisory support is insufficient, even genuine pride may fail to translate into action due to the absence of necessary external reinforcement. This moderating effect optimizes the contextual conditions for behavioral expression, amplifying the positive impact of authentic pride on proactive behavior and facilitating the effective transformation of emotional resources into tangible proactive actions (Weidman et al., 2016).

Thus, high levels of perceived supervisor support can buffer the negative effects of work anger on proactive behavior (Erdogan et al., 2011), while lower support may amplify these negative effects. Similarly, perceived supervisor support reinforces employees’ sense of self-worth and internal achievement attribution, which in turn amplifies the positive influence of authentic pride on proactive behavior.

*Hypothesis 4*: Perceived supervisor support has a moderating effect on the relationship between work anger and proactive behavior.

*Hypothesis 5*: Perceived supervisor support has a moderating effect on the relationship between authentic pride and proactive behavior.

Integration of these two moderating pathways into the previously proposed parallel mediation model led to the following hypothesis.

*Hypothesis 6*: Perceived supervisor support moderates the indirect effect of perceived overqualification on proactive behavior through work anger.

*Hypothesis 7*: Perceived supervisor support moderates the indirect effect of perceived overqualification on proactive behavior through authentic pride.

## 3 Research methodology and data analysis

### 3.1 Sample and research procedures

During the recruitment phase, we employed the “Wenjuanxing” platform to randomly recruit individuals from various industries and educational backgrounds. Utilizing the platform’s targeted sampling function, participants were selected based on the criteria of having signed their first formal labor contract post-graduation. Stratified random sampling was conducted with different proportions set according to education level and industry. In the final step, samples that did not meet the selection criteria were excluded. Additionally, to mitigate common method bias, a multi-wave data collection design was employed. The initial data collection commenced in October 2022, followed by a second round of data collection 1 month later in November 2022 to supplement the results. The final data collection was completed by the end of November 2022, with all questionnaires being independently completed by eligible respondents throughout the entire survey process. A total of 851 questionnaires were obtained, of which 695 questionnaires were valid, with a recovery validity rate of 82%.

### 3.2 Measurement of variables

All items of the questionnaire were assessed using a six-point Likert scale, with scores ranging from 1 to 6 representing very non-conformant, relatively non-conformant, somewhat non-conformant, somewhat conformant, relatively conformant, and very conformant, respectively.

Perceived overqualification was measured using the Perceived Overqualification Scale developed by Maynard et al. (2006), which measures perceived overqualification in terms of education, experience and KSA. Work anger was measured using the Work Anger Scale developed by Spielberge. Authentic pride was measured using the Authentic Pride subscale of the Authentic Pride and Arrogant Pride Scale developed by Tracy and Robins (2007). Proactive behavior was measured using the Proactive Behavior Scale developed by Yong Su and Tianjian Li tailored to the local context. Supervisor support was measured using the Supervisor Support Scale developed by Oldham and Cummings (1996). The Proactive Personality Scale was used, developed by Parker (1998). The study was statistically analyzed using SPSS 26.0 software and the PROCESS macro program.

Firstly, we conducted reliability and validity tests on the data. Exploratory factor analysis (EFA) was performed to assess both the convergent and discriminant validity of the scale. The analysis extracted the principal components as constructs, resulting in five factors corresponding to the variables. Table 1 presents the results of the factor analysis conducted using SPSS software. Table 2 reports the findings of the convergent validity and reliability analysis: the Average Variance Extracted (AVE) values exceeded the recommended threshold of 0.5, and the Composite Reliability (CR) values were above the commonly accepted standard of 0.7, indicating that all convergent validity criteria were met. Furthermore, Cronbach’s alpha coefficients exceeded 0.7, confirming the model’s acceptable internal consistency.

**Table 1:** Exploratory factor analysis of pilot data.

Constructs	Item	Component				
		1	2	3	4	5
PO: Perceived overqualification	PO1	−0.082	0.747	0.022	−0.070	−0.003
	PO2	−0.049	0.799	0.102	−0.049	−0.011
	PO3	0.002	0.748	0.126	−0.155	−0.010
	PO4	−0.076	0.794	0.143	−0.041	−0.100
	PO5	−0.013	0.820	0.101	−0.055	−0.054
	PO6	−0.054	0.716	0.091	−0.063	0.079
	PO7	−0.017	0.731	0.095	−0.031	−0.075
WA: Work anger	WA1	−0.149	0.201	0.849	−0.070	−0.047
	WA2	−0.100	0.153	0.931	−0.077	−0.060
	WA3	−0.085	0.174	0.910	−0.017	−0.094
	WA4	−0.112	0.136	0.932	−0.058	−0.086
AP: Authentic pride	AP1	0.136	−0.019	0.035	0.720	0.257
	AP2	0.170	−0.079	−0.086	0.736	−0.048
	AP3	0.158	−0.150	−0.114	0.813	0.187
	AP4	0.146	−0.133	−0.048	0.776	0.269
	AP5	0.191	−0.087	−0.028	0.827	0.178
PB: Proactive behavior	PB1	0.156	−0.045	−0.187	0.146	0.702
	PB2	0.213	−0.022	0.032	0.157	0.781
	PB3	0.241	−0.039	0.024	0.165	0.774
	PB4	0.236	0.029	−0.146	0.084	0.680
	PB5	0.213	−0.074	−0.031	0.211	0.773
SS: Perceived supervisor support	PSS1	0.759	0.041	−0.099	0.076	0.163
	PSS2	0.777	−0.021	−0.126	0.089	0.244
	PSS3	0.782	0.027	−0.059	0.129	0.193
	PSS4	0.760	−0.027	−0.041	0.193	0.208
	PSS5	0.838	−0.035	−0.051	0.147	0.142
	PSS6	0.741	−0.172	−0.067	0.161	0.155
	PSS7	0.763	−0.167	−0.093	0.131	0.100

The gray shading indicates that the absolute value of the factor loading is greater than 0.5.

**Table 2:** Reliability analysis, construct correlations and discriminant validity.

Constructs	Proactive behavior	Work anger	Supervisor support	Authentic pride	Perceived overqualification
Proactive behavior	0.737				
Work anger	−0.199	0.934			
Perceived supervisor support	0.512	−0.242	0.81		
Authentic pride	0.507	−0.161	0.396	0.75	
Perceived overqualification	−0.107	0.313	−0.138	−0.188	0.731
Mean	4.000	2.956	3.974	3.584	3.680
SD	1.164	1.402	1.185	1.175	1.471
AVE	0.543	0.873	0.656	0.562	0.534
CR	0.914	0.965	0.930	0.896	0.910
CA	0.894	0.951	0.912	0.866	0.892

### 3.3 Common method bias test

**Figure 1:**
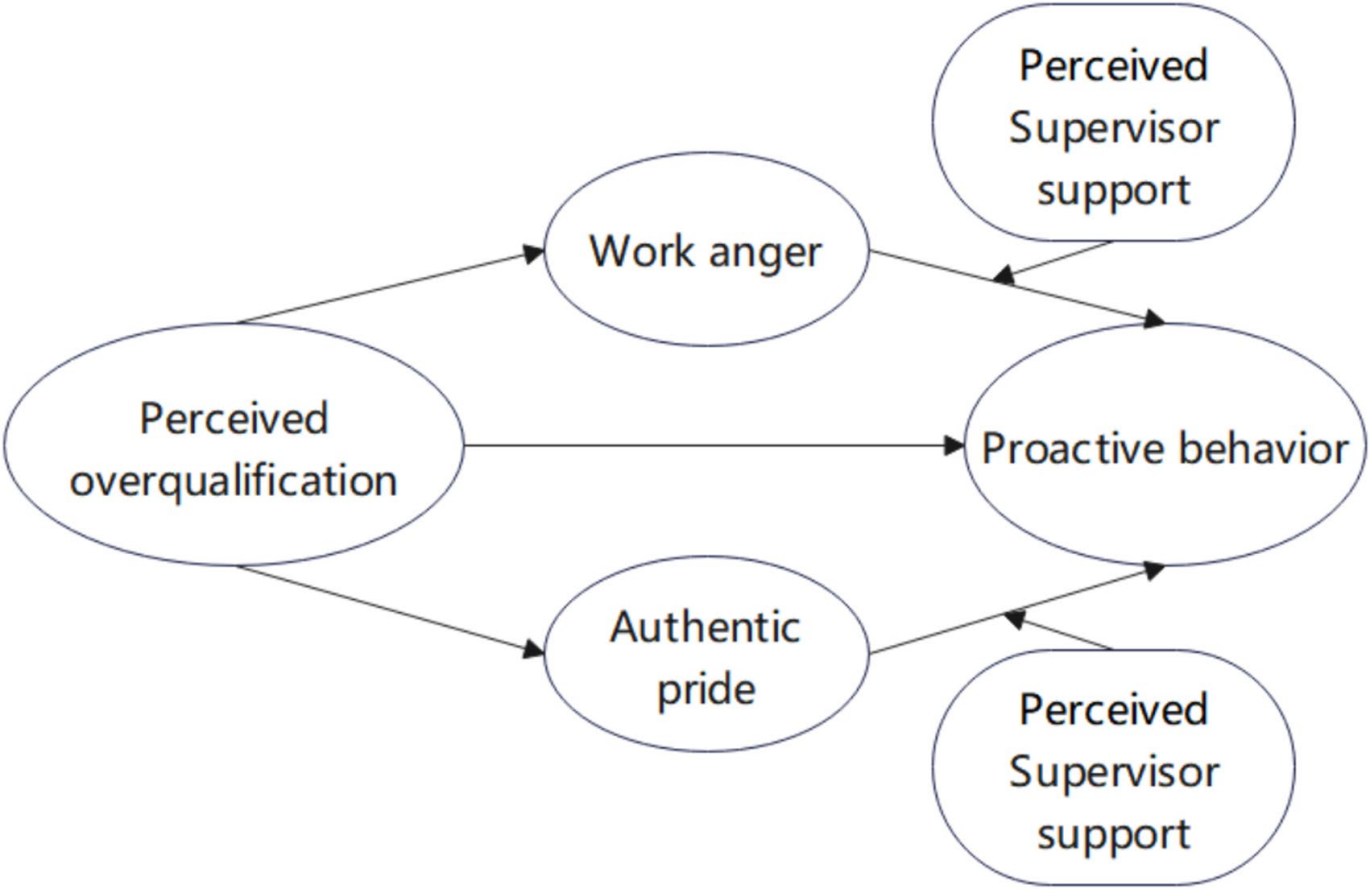
Research model.

The measurement instrument used in this study is the self-stated scale, and the data come from the same subjects. To mitigate potential common method bias affecting the results of the data analysis, Harman’s one-way test was used to test the problem of common method bias, and exploratory factor analysis was conducted on all the scale questions together. The results showed that the explained rate of variation for the first factor was 23.812% (less than 40%), indicating that the common method bias in this study was not serious. To further validate the findings, we employed AMOS software and utilized the ULMC method for common method bias testing. During the model construction process, a “common method factor” variable was introduced, and comparisons were made between the original model and the bifactor model. The results showed that the chi-square-to-df ratio changed from 4.788 to 4.804, the RMR increased from 0.094 to 0.101, the RMSEA remained at 0.074, the CFI remained at 0.926, and the TLI remained at 0.915. The data analysis revealed that the change in the chi-square/df ratio was not significant (Δ = 0.016 < 0.1); the increase in RMR was 0.007, which is below the threshold of 0.05; and no significant changes were observed in other key indices (RMSEA, CFI, TLI), suggesting that there is no substantial common method bias (Figure 1).

### 3.4 Test of the relationship between perceived overqualification and proactive behavior

Hypothesis 1 posits that perceived overqualification negatively affects proactive behavior, which was tested via multiple hierarchical regression analysis. The process of constructing the regression equation is as follows: in Model 1, the six control variables of gender, age, education, first job tenure, nature of workplace, whether currently at 1st Job, and proactive behavior is used as independent variables. In Model 2, the sense of perceived overqualification is added to the regression equation. Subsequently, SPSS 26.0 software was used to conduct multiple hierarchical regression analysis. The results in Table 3 show that the two control variables, division age and proactive personality, acted as interferences in the model. After controlling for the two interferences, perceived overqualification significantly negatively affected proactive behavior (*β* = −0.056, SE = 0.028, *p* < 0.05). Hypothesis 1 is valid.

**Table 3:** Hierarchical regression analysis of perceived overqualification on proactive behavior.

Variables	Proactive behavior			
	Model 1		Model 2	
	*β*	*SE*	*β*	*SE*
Gender	0.043	0.063	0.04	0.063
Age	0.035	0.038	0.032	0.038
Education	0.003	0.057	0.016	0.057
First job tenure	0.068**	0.023	0.068**	0.023
Nature of workplace	0.003	0.016	0.003	0.016
Currently at 1st Job or not	0.13	0.074	0.139	0.075
Proactive personality	0.432***	0.04	0.431***	0.04
Perceived overqualification			−0.056*	0.028
*R* ^2^	0.166		0.171	
Δ*R*^2^	0.166		0.005	
*F*	22.782***		20.208***	

^**^p < 0.01, ^***^p < 0.001; Parameters are non-standardized.

### 3.5 Parallel mediation effect tests for work anger and authentic pride

In this study, the mediating roles of work anger and authentic pride in the relationship between perceived overqualification and proactive behavior were analyzed after standardizing the predicted variables using the PROCESS macro program. According to the regression analysis (Table 4), perceived overqualification had a direct positive predictive effect on work anger (*β* = 0.306, *p* < 0.001), and perceived overqualification had a direct negative predictive effect on authentic pride (*β* = −0.128, *p* < 0.001). When perceived overqualification, work anger, and authentic pride simultaneously predicted proactive behavior, work anger (*β* = −0.106, *p* < 0.01) was a significant negative predictor of proactive behavior, authentic pride (*β* = 0.403, *p* < 0.001) was a significant positive predictor of proactive behavior, and perceived overqualification was not a significant direct predictor of proactive behavior.

**Table 4:** Mediation model test for work anger and authentic pride.

Regression equation		Fit indices			Coefficient significance	
Dependent variable	Independent variables	*R*	*R* ^2^	*F*	*β*	*t*
Work anger		0.371	0.138	13.692***		
					0.051	0.673
					−0.172	−3.797***
					−0.093	−1.374
					0.059	2.188*
					0.041	2.155*
					0.085	0.952
					−0.102	−2.832**
					0.306	8.549***
Authentic pride		0.352	0.124	12.116***		
	Gender				−0.02	−0.267
	Age				−0.068	−1.482
	Education				0.142	2.076*
	First job tenure				0.063	2.322*
	Nature of workplace				0.013	0.671
	Currently at 1st Job or not				−0.020	−0.221
	Proactive personality				0.302	8.367***
	Perceived overqualification				−0.128	−3.561***
Proactive behavior		0.573	0.329	33.469***		
	Gender				0.06	0.9
	Age				0.046	1.145
	Education				−0.048	−0.795
	First job tenure				0.061	2.522*
	Nature of workplace				0.002	0.126
	Currently at 1st Job or not				0.180	2.275*
	Proactive personality				0.243	7.298***
	Perceived overqualification				0.013	0.382
	Work anger				−0.106	−3.123**
	Authentic pride				0.403	11.988***

^*^p < 0.05, ^**^p < 0.01, ^***^p < 0.001.

**Figure 2:**
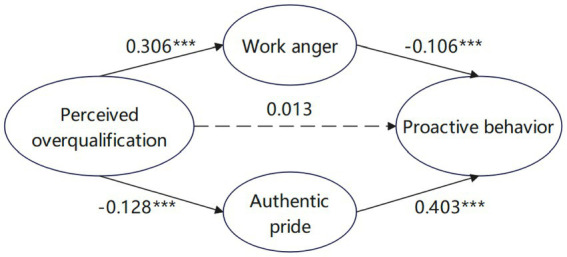
Parallel intermediation diagram. ***indicate the significance of the hypotheses.

A test of Bootstrap-based path analysis showed (see Table 5) that the mediating effects of work anger and authentic pride were significant, with a total mediating effect value of −0.084. Specifically, the indirect effect (*β* = −0.032), consisting of perceived overqualification leading to work anger, which in turn results in proactive behavior, had a 95% confidence interval of [−0.059, −0.007], excluding 0. This indicates that the mediating effect of work anger is significant. The proportion of indirect effect 1 in the total mediating effect is 38.5%, indicating that 38.5% of the influence of perceived overqualification on proactive behavior through the parallel mediating path is through the path of work anger. Indirect effect 2 (−0.052) consisting of perceived overqualification → authentic pride → proactive behavior, with a 95% confidence interval of [−0.085, −0.020], excluding 0, indicating that the mediating effect of authentic pride was significant. Indirect effect 2 accounted for 61.5% of the total mediating effect, indicating that 61.5% of the effect of perceived overqualification on proactive behavior through parallel mediating paths worked through the authentic pride path. Comparison of indirect effect 1 with indirect effect 2 reveals a 95% confidence interval of [−0.018, 0.060] containing 0. This indicates that there is no significant difference between the two mediating paths, that the mediating effects of work anger and authentic pride are comparable. So, parallel mediation is established, and Hypothesis 2 and 3 are both validated. The specific path of perceived overqualification acting on proactive behavior is shown in Figure 2.

**Table 5:** Analysis of mediating effects of work anger, authentic pride.

Path	Effect size	Proportion of total mediation	Lower BootCI	Upper BootCI
Ind1: perceived overqualification → work anger → proactive behavior	−0.032	38.50%	−0.059	−0.007
Ind2: perceived overqualification → authentic pride → proactive behavior	−0.052	61.50%	−0.085	−0.02
Total mediation effect: Ind1 + Ind2	−0.084	100%	−0.129	−0.04
Comparative Mediation Effect: Ind1 − Ind2	0.019		−0.018	0.06

Boot CI is 95% Confidence Interval.

### 3.6 Moderating effect test of perceived supervisor support

Based on the mediation effect test, this study employed Model 14 in the PROCESS macro program to examine the moderating role of perceived supervisor support in the relationship between perceived overqualification, work anger, authentic pride and proactive behavior. The results showed (Table 6) that after putting the sense of supervisor support into the model, the product term of work anger and the sense of supervisor support had a significant predictive effect on proactive behavior (*β* = 0.071, *p* < 0.01). This indicated that the sense of supervisor support was able to modulate the predictive effect of work anger on proactive behavior. The predictive effect of the product term of authentic pride and sense of supervisor support on proactive behavior was not significant (*β* = −0.020, *p* = 0.440), suggesting that sense of supervisor support does not moderate the effect of authentic pride on proactive behavior. Thus, Hypothesis 4 is valid and Hypothesis 5 is not valid.

**Table 6:** Moderating role tests for perceived supervisor support.

Regression equation		Fit indices			Coefficient significance	
Dependent variable	Independent variables	*R*	*R* ^2^	*F*	*β*	*t*
Proactive behavior		0.656	0.430	39.503***		
	Gender				0.052	0.843
	Age				0.079	2.090*
	Education				−0.034	−0.604
	First job tenure				0.047	2.091*
	Nature of workplace				−0.013	−0.797
	Currently at 1st Job or not				0.144	1.970*
	Proactive personality				0.214	6.772***
	Perceived overqualification				0.022	0.701
	Work anger				−0.041	−1.282
	Authentic pride				0.294	8.993***
	Perceived overqualification				0.337	10.222***
	Work anger × Perceived overqualification				0.071	2.802**
	Authentic pride × Perceived overqualification				−0.02	−0.772

^*^p < 0.05, ^**^p < 0.01, ^***^p < 0.001.

**Figure 3:**
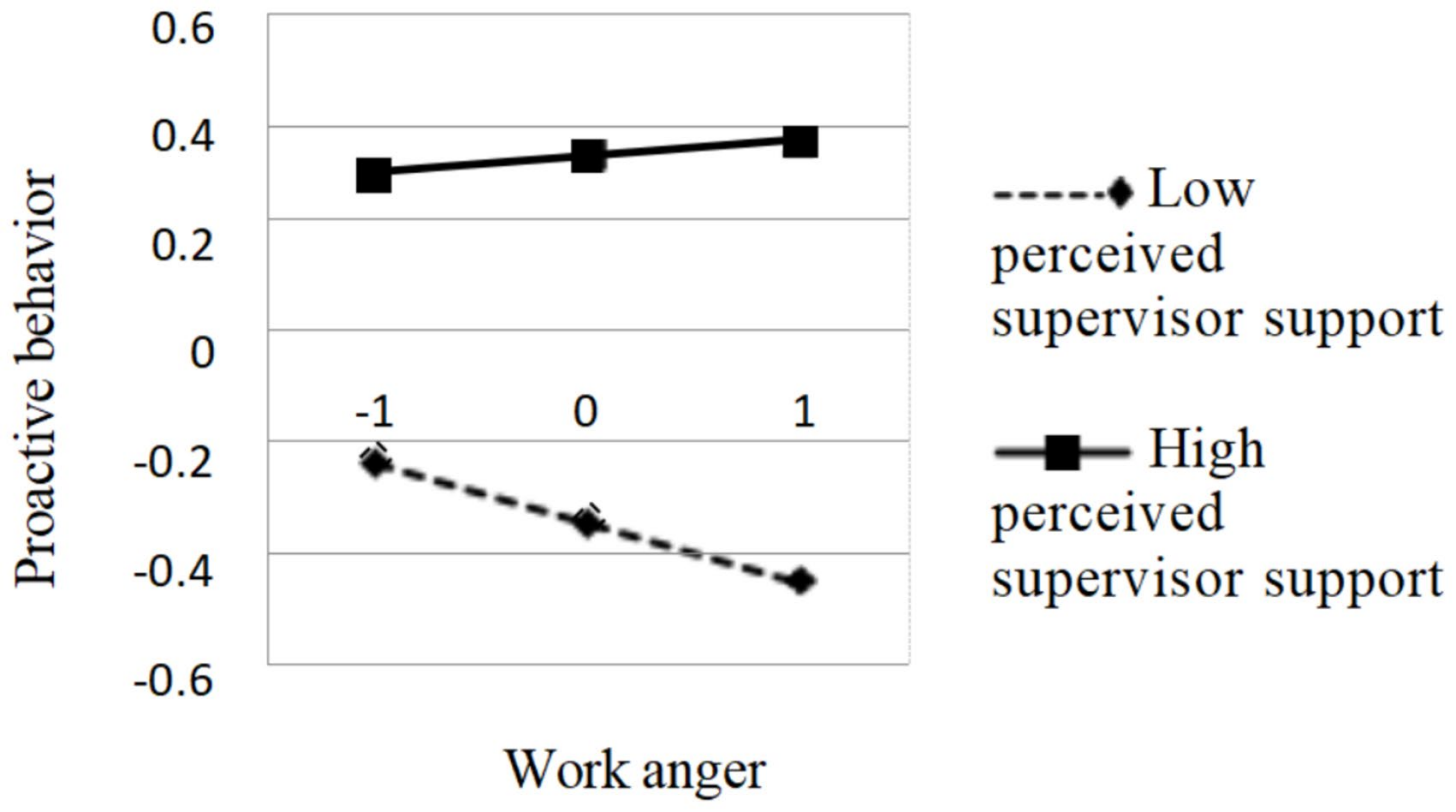
Moderating effect of perceived supervisor support on the relationship between work anger and proactive behavior.

To further illustrate the moderating effect of supervisor support on the second half of the mediating effect of “perceived overqualification → work anger → proactive behavior,” the author divided the group into two subgroups according to the standardized scores of supervisor support (*Z* ≤ −1SD) and high subgroups (*Z* ≥ 1SD) to examine the effects of work anger on proactive behaviors at different levels of supervisor support. The interaction is shown in Figure 3. Simple slope analyses indicated that work anger was a significant negative predictor of proactive behavior for individuals with low levels of supervisor support (M − 1SD), with significant decreases in proactive behavior as work anger increased, simple slope = −0.11, *t* = −2.71, *p* < 0.01. Whereas, for individuals with high levels of supervisor support (M + 1SD), work anger was not a significant predictor of proactive behavior, simple slope = 0.03, t = 0.74, *p* = 0.46. Overall, the effect of work anger on proactive behavior is influenced by the level of supervisor support.

**Figure 4:**
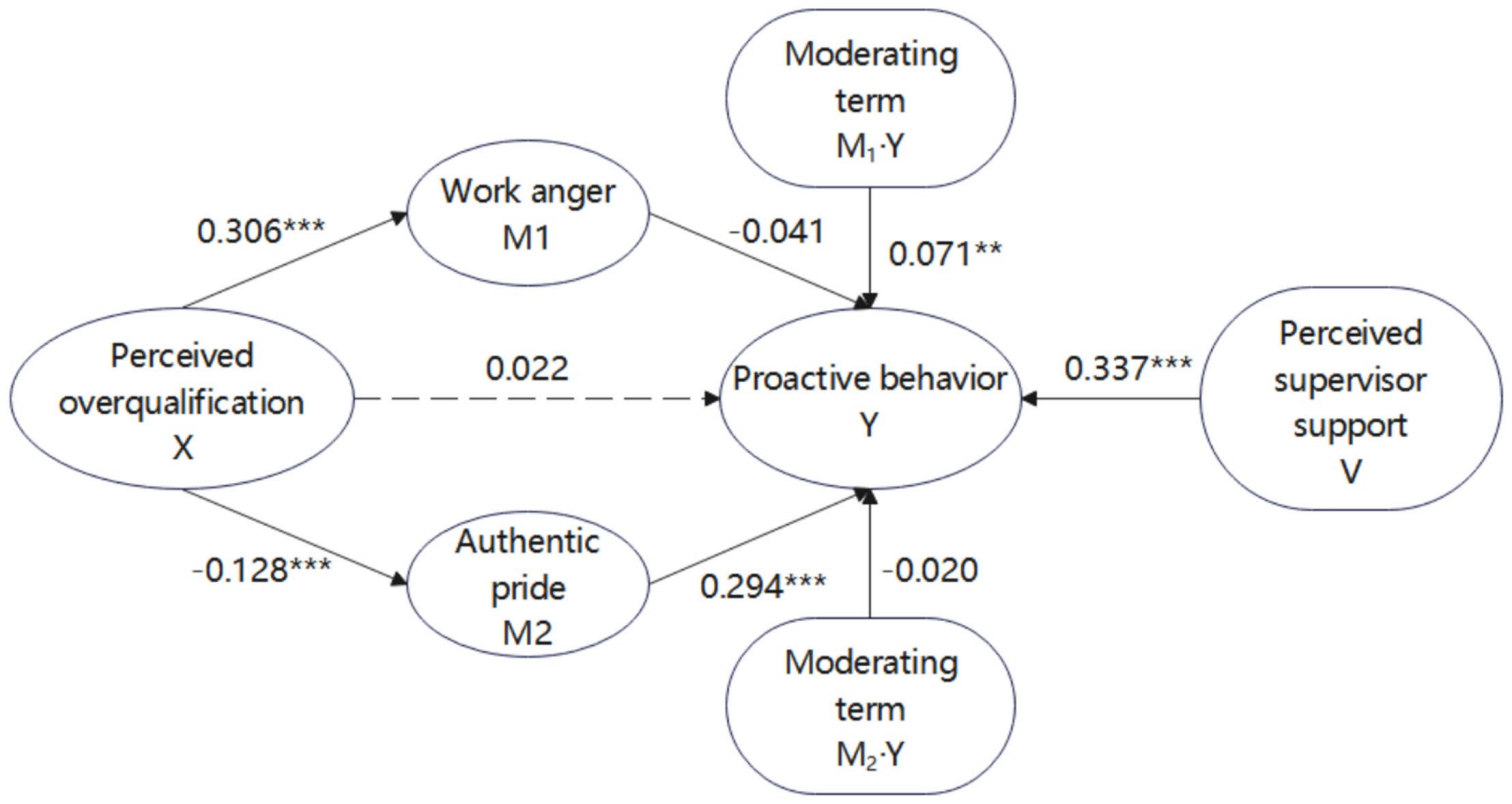
Plot of the moderating effect of perceived supervisor support. ***indicate the significance of the hypotheses.

The mediating role of supervisor support in the path of “perceived overqualification → work anger → proactive behavior” is further examined. As shown in Appendix Table 5, the mediating effect of perceived overqualification on proactive behavior through work anger was −0.034 at a low level of supervisor support (M − 1SD), with a 95% confidence interval of [−0.066, −0.006], excluding 0. Also, the mediating effect was significant. The mediating effect of work anger on perceived overqualification and proactive behavior was not significant at a high level of supervisor support (M + 1SD). The mediating effect was not significant. That is, perceived overqualification affects proactive behavior through work anger only when the level of supervisor support is low. Overall, the path of perceived overqualification’s influence on proactive behavior through work anger is affected by the high level of supervisor support, indicating that the moderating effect of supervisor support is significant in the mediating effect of work anger on the relationship between perceived overqualification and proactive behavior. Hypothesis 6 is verified. The moderating effect is shown in Figure 4.

## 4 Discussion and implications

### 4.1 Main findings

Drawing on the proactive motivation model and social cognitive theory, this study examined the relationship between perceived overqualification and proactive behavior among first-time employees, explored the mediating roles of work anger and authentic pride, and further investigated the boundary conditions of this process. The empirical results revealed the following findings:

(1) In the context of employees’ first job, perceived overqualification significantly and negatively predicts proactive behavior. The higher the level of perceived overqualification, the less proactive behavior employees’ exhibit.
(2) Work anger and authentic pride play parallel mediating roles in the relationship between perceived overqualification and proactive behavior. Specifically, perceived overqualification indirectly and negatively affects proactive behavior through increased work anger and decreased authentic pride.
(3) Perceived supervisor support serves as a significant moderating factor in the relationship between perceived overqualification and proactive behavior. First, perceived supervisor support moderates the relationship between work anger and proactive behavior: the negative predictive effect of work anger on proactive behavior is significant only when perceived supervisor support is low, and becomes nonsignificant when perceived supervisor support is high. Second, perceived supervisor support moderates the indirect effect of perceived overqualification on proactive behavior through work anger. The mediating effect of anger is significant only among employees with low levels of perceived supervisor support, whereas it weakens when supervisory support is high.

### 4.2 Theoretical implications

Focusing on first-time employees, this study explores how perceived overqualification influences proactive behavior during the initial stage of one’s career, and—drawing upon the proactive motivation model—reveals the underlying emotional transmission mechanism and contextual moderating effects. The findings demonstrate a significant negative relationship between perceived overqualification and proactive behavior among employees in their first job. This result provides empirical support for Erdogan’s argument that perceived overqualification can exert a detrimental effect on proactive behavior, further clarifying previous inconsistencies regarding the functional outcomes of perceived overqualification (Erdogan et al., 2020). Moreover, it extends the application of the proactive motivation model to the early stage of career development.

First, at the theoretical level, this study reveals the underlying psychological mechanisms through which perceived overqualification affects proactive behavior, by examining the distinct mediating roles of different types of emotions. The findings indicate that perceived overqualification indirectly influences proactive behavior through two distinct emotional pathways—work anger and authentic pride. In other words, employees who perceive themselves as overqualified tend to experience negative emotions such as anger and frustration due to feelings of being undervalued or unrecognized, which in turn diminish their willingness to engage proactively. Meanwhile, perceived overqualification suppresses employees’ authentic pride, making it difficult for them to derive a sense of achievement and self-worth from their work, thereby further inhibiting the emergence of proactive behavior. This finding empirically demonstrates the existence of the affective pathway within the proactive motivation model, addresses a specific gap regarding the role of discrete emotions in the link between perceived overqualification and proactive behavior within this model (Qiao and Yang, 2021) and enriches the theoretical system of the proactive motivation model. Additionally, the analysis of the parallel mediating effects of work anger and authentic pride responds both to Ilies’s call for investigations into the impact of distinct emotions on work behaviors (Ilies et al., 2013) and to Johnson’s earlier reference to perceived overqualification, highlighting “the need for further research on the joint role of positive and negative emotions in the workplace and the mediating function of these emotions” (Johnson and Johnson, 2000).

Second, this study redefines and refines the role of perceived supervisor support within the mechanism linking perceived overqualification to proactive behavior, thereby enriching the boundary conditions of overqualification research. By introducing perceived supervisor support—a key situational factor and an antecedent of perceived organizational support—this study further clarifies how organizational support factors function within the “perceived overqualification–emotion–behavior” system. The empirical results demonstrate that perceived supervisor support significantly moderates the indirect effect of perceived overqualification on proactive behavior via work anger. Specifically, when employees perceive low supervisor support, perceived overqualification is more likely to elicit work anger, which in turn reduces proactive behavior. Conversely, when perceived supervisor support is high, this negative indirect effect is substantially alleviated. Meanwhile, the hypothesized moderating effect of perceived supervisor support on the “perceived overqualification–authentic pride–proactive behavior” pathway was not supported. This suggests that authentic pride primarily stems from individuals’ intrinsic evaluation of task value and personal effort, and is less sensitive to external support conditions. Specifically, according to Self-Determination Theory, when individuals experience authentic pride, they tend to attribute their success to their own competence and autonomy, which are core sources of intrinsic motivation (Ryan and Deci, 2000). From the perspective of the structure of pride, classical emotion theory—particularly the two-facet model of pride proposed by Tracy and Robins—also supports this view: authentic pride is elicited by internal, unstable, and controllable attributions (e.g., one’s own effort or behavior) (Tracy and Robins, 2007). In other words, when individuals perceive that their achievements are the result of their own efforts rather than external imposition or support, they are more likely to experience authentic pride rather than arrogant pride. Therefore, the non-significance of H5 in our study can be interpreted as follows: authentic pride may rely more heavily on individuals’ internal evaluations of their own effort and growth, rather than on responses to supervisor support or external resources. This suggests that the boundary effect of supervisor support may operate more strongly on inhibiting negative emotions rather than directly enhancing positive emotions.

Finally, this study makes three major theoretical contributions at the overall conceptual level. First, it develops and validates a “perceived supervisor support–emotion–behavior” mechanism grounded in the proactive motivation model, revealing how perceived overqualification influences proactive behavior through distinct emotional pathways, and providing an integrated framework for understanding the psychological transmission process of perceived overqualification. Second, by situating the analysis in the context of employees’ first job experience, the study highlights the cognitive and emotional vulnerability of individuals in the early stage of career socialization, thereby offering a theoretical foundation for future research examining perceived overqualification from a career-stage perspective. Third, by confirming the moderating effect of perceived supervisor support within the emotional pathways, the study extends the “situation–emotion–motivation” interaction within the proactive motivation model and underscores the boundary role of supervisory support in regulating emotion and sustaining proactive behavior. Overall, this study deepens the understanding of the negative psychological mechanisms underlying perceived overqualification, broadens the applicability of the proactive motivation model, and provides new theoretical insights for organizations seeking to foster proactive development among first-time employees.

### 4.3 Practical implications

Based on the research findings, this study proposes several practical implications at the organizational, managerial, and individual levels, aiming to help organizations more effectively stimulate new employees’ proactive potential and mitigate the negative effects of perceived overqualification.

From the organizational side, organizations should prioritize “ability–task fit” during recruitment and job placement by conducting systematic job analyses and implementing dynamic task adjustments to align new employees’ capabilities with job requirements, thereby reducing the likelihood of perceived overqualification at its source. Furthermore, organizations should establish multi-level incentive systems that incorporate proactive learning, problem-solving, and voice behaviors into performance evaluation criteria, reinforcing the organizational recognition of proactive behavior. For first-time employees, firms can design “ability-enhancing” tasks—such as project-based job rotation or cross-departmental collaboration—that not only allow them to apply existing competencies but also provide sufficient challenge to sustain the activation of authentic pride. At the same time, organizations should develop internal feedback mechanisms and psychological support programs to monitor new employees’ emotional states, alleviating the accumulation and spread of work anger in a timely manner and preventing perceived overqualification from evolving into negative emotions or withdrawal behaviors.

From a managerial perspective, supervisors should pay special attention to employees in their first job, who tend to experience emotional fluctuations due to limited work experience and a still-developing professional identity. As direct leaders and primary sources of social support, supervisors play a pivotal role in shaping newcomers’ career adaptation and proactive motivation. The findings indicate that perceived supervisor support can significantly buffer the negative indirect effect of perceived overqualification on proactive behavior through work anger. Therefore, managers should actively provide task guidance, performance feedback, and recognition-based communication to help employees interpret perceived overqualification appropriately, avoiding its misperception as neglect or wasted potential.

In practice, supervisors should first offer structural support by developing clear work plans, task goals, and performance indicators, enabling employees to understand job expectations and standards, thereby reducing anxiety and uncertainty stemming from task ambiguity. At the same time, they should ensure adequate instrumental support through fair access to work resources and transparent career development paths, reducing the sense of powerlessness and alienation that often accompanies perceived overqualification. Second, managers should strengthen emotional support by offering timely positive feedback, public recognition, and performance acknowledgment, ensuring employees feel that their efforts are visible and appreciated. Such recognition not only alleviates the emotional imbalance caused by perceived overqualification but also reinforces employees’ self-worth and effort attribution, helping to sustain and enhance authentic pride. Third, supervisors should provide developmental support through training, mentoring, and capability-building programs that create opportunities for continuous learning and growth. This enables newcomers to view their current job as a developmental stage for accumulating experience and honing competence, maintaining a positive mindset and exploratory motivation. In addition, managers should closely monitor employees’ psychological states by establishing regular communication and counseling mechanisms to identify and resolve emotional distress or cognitive biases in time, preventing the accumulation of negative emotions that may lead to withdrawal or passive behavior.

For first-time employees, properly recognizing and managing perceived overqualification is essential for long-term career development. Individuals should avoid interpreting “having higher abilities than job requirements” as professional failure and instead reframe it as an opportunity for learning and growth through self-reflection and goal reorientation. At the same time, employees should proactively cultivate emotional regulation abilities—such as cognitive reappraisal and positive self-affirmation—to mitigate the behavioral interference of negative emotions like anger and enhance the intrinsic motivational power derived from authentic pride. In doing so, individuals can achieve both personal growth in their first job and sustained career advancement through accumulated experience and continuous self-improvement.

### 4.4 Limitations and directions for future research

First, limitations of research instruments and measurements. Although this study employed well-established and widely validated scales, only the proactive behavior scale was originally developed by Chinese scholars based on local contexts. The other scales were developed in Western settings and later translated and adapted for use in China. While these instruments demonstrate satisfactory reliability and validity, their contextual applicability within the Chinese workplace may still be limited. Future research could refine or reconstruct existing measurement tools by incorporating Chinese cultural and workplace characteristics and conduct empirical studies based on localized samples to improve the accuracy and interpretability of research findings.

Second, limitations of the research model. Although this study introduced perceived supervisor support as a moderating variable, it primarily examined its moderating role in the second stage of the hypothesized model (i.e., the emotion–behavior relationship). The potential moderating effects of supervisor support in the earlier stage—specifically whether it buffers the effect of perceived overqualification on emotional responses such as anger or pride—were not explored. Future research could further test this stage of moderation to enhance the theoretical contribution of the model.

Third, limitations in data collection. (1) Sample-related limitations. Although the sample size (*N* = 695) met statistical requirements, data were collected primarily through online surveys, with participants concentrated in certain regions and industries, which may limit representativeness. Future studies could combine online and offline data collection methods, expand the sample size and diversity, and include broader geographic and industrial distributions. In addition, longitudinal designs could be employed to track changes in perceived overqualification across different stages of employment, thereby revealing the developmental trajectory and long-term effects on proactive behavior among first-time employees. (2) Limitations of cross-sectional data. While this study adopted a two-wave cross-sectional design (October and November 2022), which effectively controls for external interference and captures newcomers’ psychological states and behavioral responses at specific time points, its ability to draw causal inferences remains constrained. Due to the short interval between data collections (only 1 month), it may fail to fully reflect the dynamic interaction among perceived overqualification, emotions, and proactive behavior, nor can it rule out the possibility of reverse causal relationships. For instance, employees with low levels of proactive behavior may induce stronger perceived overqualification or more intense negative emotions, ultimately leading to interpretative bias. Furthermore, the cross-sectional design struggles to detect the long-term fluctuation characteristics of emotions and behaviors during the early career stage. Future research could employ longer-term longitudinal designs, experimental designs, or multi-source data approaches to verify causal mechanisms and enhance the robustness and external validity of the research findings.

Fourth, limitations in career stage and contextual scope. This study focused on first-time employees, emphasizing the characteristics of perceived overqualification and proactive behavior during the initial career stage. However, employees at different career stages differ substantially in motivation, self-efficacy, and organizational embeddedness. This study did not conduct subgroup comparisons across career stages, though these stages may moderate the relationship between perceived overqualification and proactive behavior. Future research could examine whether career stage or tenure moderates the cognitive and emotional mechanisms underlying perceived overqualification. For instance, long-tenured employees may interpret perceived overqualification as a stable competence advantage, whereas newcomers may view it as a sign of undervaluation or constrained development, leading to divergent emotional and behavioral outcomes. Future studies could also incorporate individual difference variables such as career development orientation or learning goal orientation to further uncover the heterogeneity of perceived overqualification mechanisms across career stages.

## Data Availability

The original contributions presented in the study are included in the article/Supplementary material, further inquiries can be directed to the corresponding author.
