# Supplementary material for: Anger or pride? The effect of overqualification on employees’ first job behavior from the perspective of the proactive motivation model
Source: Front Psychol. 2026 Jan 5;16:1682877. doi: 10.3389/fpsyg.2025.1682877 (PMC12812671; doi:10.3389/fpsyg.2025.1682877)
Supplement: Supplementary file 1 [file Supplementary_file_1.docx]

### Appendices

Table 1 Basic information of the survey sample

|  | Categories | Number | Percentage(%) |
| --- | --- | --- | --- |
| Gender | Male | 267 | 38.4 |
|  | Female | 428 | 61.6 |
| Age | ≤25years old | 98 | 14.1 |
|  | 26-30 | 353 | 50.8 |
|  | 31-35 | 162 | 23.3 |
|  | 36-40 | 60 | 8.6 |
|  | >40years old | 22 | 3.2 |
| Education | High school diploma and below | 3 | 0.4 |
|  | Junior college degree | 23 | 3.3 |
|  | Bachelor’s degree | 475 | 68.3 |
|  | Master's degree | 190 | 27.3 |
|  | Doctor's degree | 4 | 0.6 |
| First job tenure | Less than a year | 163 | 23.5 |
|  | 1-2 years | 141 | 20.3 |
|  | 2-3 years | 92 | 13.2 |
|  | 3-5 years | 148 | 21.3 |
|  | 5-10 years | 113 | 16.3 |
|  | More than 10 years | 38 | 5.5 |
| Nature of workplace | State-owned Enterprise | 157 | 22.6 |
|  | Private Enterprise | 248 | 35.7 |
|  | Foreign-Invested Enterprise | 51 | 7.3 |
|  | Joint venture | 33 | 4.7 |
|  | Government Agency | 97 | 14.0 |
|  | Public Institution | 70 | 10.1 |
|  | Others | 39 | 5.6 |
| Currently at 1st Job or not | Yes | 314 | 45.2 |
|  | No | 381 | 54.8 |

Table 2 Results of descriptive statistics and correlation analysis

| Variable | 1 | 2 | 3 | 4 | 5 | 6 | 7 | 8 | 9 | 10 | 11 | 12 |
| --- | --- | --- | --- | --- | --- | --- | --- | --- | --- | --- | --- | --- |
| Gender |  |  |  |  |  |  |  |  |  |  |  |  |
| Age | -0.186** |  |  |  |  |  |  |  |  |  |  |  |
| Education | 0.033 | -0.019 |  |  |  |  |  |  |  |  |  |  |
| First job tenure | -0.088* | 0.270** | -0.063 |  |  |  |  |  |  |  |  |  |
| Nature of workplace | 0.162** | -0.113** | 0.079* | -0.005 |  |  |  |  |  |  |  |  |
| Currently at 1st Job or not | -0.099** | 0.334** | -0.127** | -0.352** | -0.193** |  |  |  |  |  |  |  |
| Proactive personality | -0.099** | 0.050 | 0.030 | 0.004 | -0.097* | 0.071 |  |  |  |  |  |  |
| Perceived overqualification | -0.025 | -0.013 | 0.110** | -0.029 | -0.011 | 0.044 | -0.009 |  |  |  |  |  |
| Work anger | 0.056 | -0.143** | -0.021 | 0.026 | 0.095* | -0.049 | -0.121** | 0.301** |  |  |  |  |
| Authentic pride | -0.026 | -0.025 | 0.069 | 0.087* | 0.010 | -0.064 | 0.301** | -0.125** | -0.134** |  |  |  |
| Proactive behavior | -0.037 | 0.111** | -0.004 | 0.108** | -0.046 | 0.067 | 0.382** | -0.075* | -0.191** | 0.488** |  |  |
| Perceived  supervisor Support | -0.021 | -0.039 | 0.014 | 0.047 | 0.050 | -0.024 | 0.221** | -0.136** | -0.243** | 0.383** | 0.504** |  |
| M | 1.620 | 2.360 | 3.240 | 3.030 | 3.040 | 1.550 | 4.303 | 3.680 | 2.956 | 3.584 | 4.000 | 3.974 |
| SD | 0.487 | 0.936 | 0.537 | 1.581 | 1.917 | 0.498 | 0.746 | 1.083 | 1.310 | 0.877 | 0.854 | 0.960 |

a. ** ：P < 0.01 ；* ：P < 0.05

Table3 Mediation test with moderation (work anger as mediating variable)

|  | Perceived  supervisor Support | Effect size | *BootSE* | Lower *BootCI* | Upper *BootCI* |
| --- | --- | --- | --- | --- | --- |
| The mediating role of work anger | 3.014(M-1SD) | -0.034 | 0.016 | -0.066 | -0.006 |
|  | 3.974(M) | -0.013 | 0.011 | -0.034 | 0.01 |
|  | 4.934(M+1SD) | 0.009 | 0.016 | -0.019 | 0.042 |
